# Structural modularity and evolutionary dynamics of the ABC transporter protein family

**DOI:** 10.1093/bib/bbaf631.018

**Published:** 2025-12-12

**Authors:** Ichda Arini Dinana, Yukihiko Kubota, Masahiro Ito

**Affiliations:** Advanced Life Sciences Program, Graduate School of Life Sciences, Ritsumeikan University, Japan; Department of Bioinformatics, College of Life Sciences, Ritsumeikan University, Japan; Advanced Life Sciences Program, Graduate School of Life Sciences, Ritsumeikan University, Japan

## Abstract

**Aim:**

ATP-binding cassette (ABC) transporters are among the most functionally diverse protein superfamilies, essential for transmembrane substrate transport and implicated in various diseases, including cancer and cystic fibrosis [1]. While previous studies have focused on conserved nucleotide-binding domains (NBDs) and transmembrane domains (TMDs) [2], the functional and evolutionary significance of non-canonical regions, particularly intrinsically disordered linkers, remains poorly understood.

**Methods:**

A comprehensive computational pipeline were applied to analyze the domain organization, structural disorder, selection pressure, and post-translational modification (PTM) patterns across ABC transporter subfamilies. Phylogenetic trees were constructed from aligned protein sequences. Codon-based evolutionary tests were conducted using the HyPhy package (FEL and MEME). Structural disorder and secondary structure were predicted using AIUPred and NetSurfP, respectively. Structural transitions were inferred using GLOOME and PTM sites were predicted with MusiteDeep.

**Results:**

Linker regions in full transporters were highly disordered, enriched in predicted PTMs, and subject to episodic positive selection. While conserved domains exhibited subfamily-specific divergence and structural rigidity. These findings suggest that linker regions may function as flexible modules with adaptive and regulatory potential.

**Conclusion:**

Our findings indicate that, within the ABC transporter structure, besides conserved domains, linker regions also play crucial roles by combining structural dynamics with regulatory control, offering new insight into how modular evolution drives their functional diversification and structural complexity.

**References:**

1. Dvorak P., Pesta M., Soucek P. ‘ABC gene expression profiles have clinical importance and possibly form a new hallmark of cancer.’ *Tumor Biology* 2017;39(5):1010428317699800.

2. Rees D.C., Johnson E., Lewinson O. ‘ABC transporters: the power to change.’ *Nature Reviews Molecular Cell Biology* 2009;10(3):218–227.

